# Enigma of medial coronary artery calcifications: an unfolding story

**DOI:** 10.1093/ehjopen/oeag093

**Published:** 2026-07-08

**Authors:** Peter Lanzer

**Affiliations:** Middle German Heart Center, Department of Internal Medicine, Goitzsche Klinik, Friedrich-Ludwig-Jahn-Straße 2, Bitterfeld 04769, Germany

**This editorial refers to ‘Pathology-validated prevalence and clinical characteristics of coronary medial arterial calcification’, by Y. Matsumoto *et al.*, https://doi.org/10.1093/ehjopen/oeag076**

Medial arterial calcification (MAC) is a systemic vascular disorder characterized by the accumulation of hydroxyapatite (HAP) with a strong predilection for the peripheral arteries and a remarkable association with aging and diseases such as diabetes and chronic kidney disease (CKD).^1^

Although MAC in the coronary arteries was reported as early as 1977,^2^ a systematic examination of the coronary artery media is lacking. In fact, it has been tacitly and explicitly assumed that all coronary artery calcifications (CAC) are intimal and atherosclerosis based.^3^

In a pivotal 1995 study, Mintz and colleagues demonstrated using intravascular ultrasound (IVUS) that CAC can be topographically classified as superficial, deep, or both. Superficial calcifications were defined as deposits located closer to the intimal–lumen interface than to the adventitia, while deep calcifications were defined as deposits at the media–adventitia border or closer to the adventitia than to the lumen.^4^ Using this topographic distinction, the authors found that 48% were only superficial, 28% only deep, and 24% combined.^4^ The authors acknowledged the limitations of IVUS in defining CAC owing to acoustic shadowing and the poor delineation of the coronary artery internal and external elastic membranes (IEL and EEL), limitations shared by optical coherence tomography (OCT).^5^

Given these limitations of high-resolution intracoronary imaging techniques for visualizing CAC, systematic histological examinations of human coronary artery specimens remain indispensable for a rigorous characterization of CAC.

In this issue of the *European Heart Journal Open*, Matsumoto and colleagues report the results of histological analysis of 4508 histologic sections from 327 coronary arteries in 112 autopsy cases from a multicentre registry.^6^ The main findings were that MAC, defined as calcium within the media between the IEL and EEL, was rare, 44/4508 sections (1.0%), 7/327 arteries (2.1%), and 4/112 patients (3.6%), and occurred exclusively in the terminal stages of CKD. In contrast, deep intimal calcification (DIC), defined as intimal calcium adjacent to the IEL and occupying <20% of total intimal thickness, was found in 111/4508 sections (2.5%), 58/327 arteries (17.7%), and 40/112 patients (35.7%).^6^ Finally, advanced intimal atherosclerosis was present in 2996/4508 (66.5%) sections; 74.2% were sheet calcification and 25.8% were nodular calcification.^6^ Based on these findings, the authors propose a new classification for deep coronary calcification, comprising three distinct phenotypes: (i) deep intimal calcification (DIC), (ii) secondary nodular protrusion, and (iii) rare true Mönckeberg-type isolated sheets.

**Figure 1:**
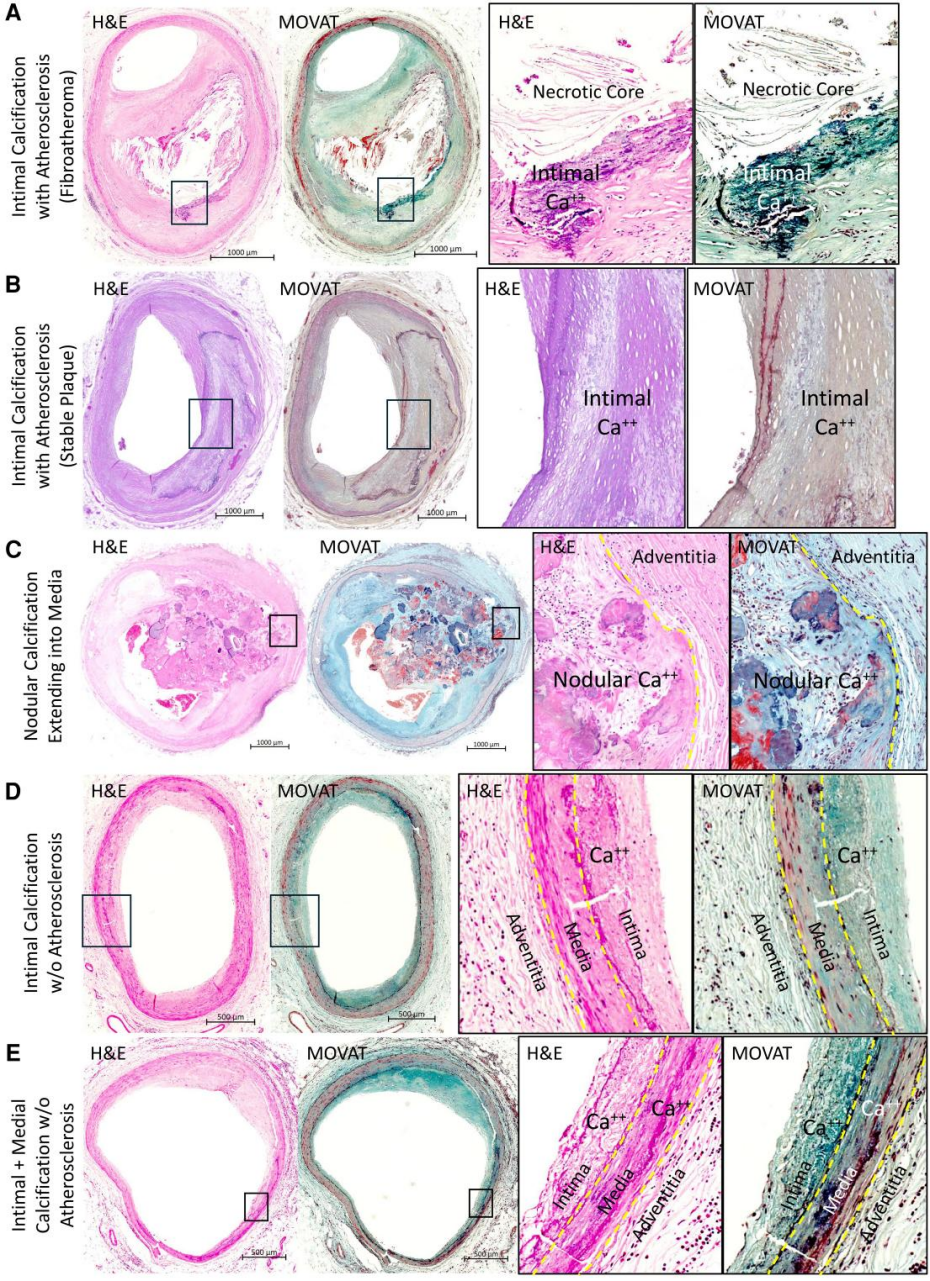
(*A*) Intimal calcification associated with atherosclerosis in a fibroatheroma lesion (typical). Calcification is localized to the outer rim of the necrotic core. (*B*) Intimal calcification associated with atherosclerosis in a stable plaque (typical). Sheet calcification is observed near the luminal surface. (*C*) Nodular calcification extending into the media, originating from intimal calcification. The internal elastic lamina is focally absent. (*D*) Intimal calcification without atherosclerosis (atypical pattern seen predominantly in chronic kidney disease). (*E*) Combined intimal and medial calcification without atherosclerosis (atypical pattern seen predominantly in chronic kidney disease). Isolated medial calcification is observed mainly in genetic abnormalities. H&E, hematoxylin and eosin staining; Movat, Movat pentachrome staining; Ca++, calcification. For each panel, the left images represent low-power views and the right, black-boxed images represent high-power views (courtesy of Dr Renu Virmani and Dr Tomoyo Hamana, CVPath Institute, Gaithersburg, MD, USA).

These findings confirm that typical Mönckeberg (MAC) lesions—that is, calcifications strictly confined to the media—are uncommon in the studied population and constitute a meaningful contribution to our understanding of CAC pathogenesis. Nevertheless, the authors’ decision to assign DIC to the atheroma-based, intimal CAC framework warrants further scrutiny. In the peripheral arteries, MAC frequently manifests as calcifications of the IEL itself, and the IEL calcifications are widely regarded as an early phenotypic expression of MAC that is independent of atherosclerosis. Accordingly, the classification of IEL calcifications as intimal, as proposed by the authors, may require further detailed re-examination in the light of the distinctive structural characteristics of the coronary IEL, which, compared with the IEL in other vascular beds, is thinner, and frequently discontinuous and fragmented. Targeted histological analysis of the spatial coincidence between coronary IEL calcifications and intimal atheroma would help correctly assign them to the respective coronary artery wall layers. The presence of coronary artery IEL calcifications in the absence of coronary artery atherosclerosis could suggest a medial origin of these lesions. Furthermore, given the high phenotypic plasticity and migratory capacity of coronary smooth muscle cells (SMC) compared with SMC from other vascular beds, atheroma may represent a critical, but not the exclusive, factor in intimal CAC (*Figure 1*).
